# Anxiety, repetitive and restricted behaviors and interests, and social communication in autistic adults: an exploratory analysis of a phase 3, randomized clinical trial

**DOI:** 10.1038/s41598-025-22659-y

**Published:** 2025-11-06

**Authors:** Eduardo A. Aponte, Julian Tillmann, Teresa Gleissl, Marta del Valle Rubido, Lorraine Murtagh, Kevin Sanders, Christopher H. Chatham, Thomas Wiese, Eugénie E. Suter

**Affiliations:** 1 Roche Pharma Research and Early Development, Roche Innovation Center Basel, F. Hoffmann-La Roche Ltd., Basel, Switzerland; 2 Global Product Development Neuroscience, F. Hoffmann-La Roche Ltd., Basel, Switzerland; 3 Product Development Neuroscience, Genentech, Inc., South San Francisco, CA USA

**Keywords:** Autism, Anxiety, Repetitive behaviors, Socialization, Communication, Anxiety, Autism spectrum disorders

## Abstract

Autistic adults are highly vulnerable to mental health problems and yet, our understanding of co-occurring psychiatric disorders in this population is limited. Anxiety is one of the most pervasive psychiatric disorders that affects autistic adults. Here, we investigated the association between anxiety, restricted and repeated behaviors and interests (RRB), and challenges in social communication and interaction (CSCI) as a post-hoc analysis of a large Phase 3 clinical trial (NCT03504917). The study enrolled 322 adults (64 females, age 27 ± 10) assessed at baseline and weeks 12, 24, 36, and 52, with the Hamilton Anxiety Rating Scale, the Repetitive Behaviors Scale – Revised, and the Vineland-II for CSCI. All analyses were blind to treatment assignment as the primary study analysis had found no treatment effects. Anxiety levels were significantly correlated with RRB and CSCI at baseline (RRB: *r* = 0.19, *P* < 10^–3^; CSCI=–0.13, *P* = 0.02) and across the entire study (RRB: *r* = 0.22, *P* < 10^–3^; CSCI=–0.16, *P* < 0.01). However, a mediation analysis revealed that the effect of CSCI on anxiety was fully explained by RRB (*P* = 0.17). While no causal relationship between both symptom domains has been established yet, our findings suggest that anxiety symptoms are associated with increased RRB, warranting further exploration of a potential causal association and implications for treatment.

**Clinical trial registration**: The research presented is registered at ClinicalTrials.gov with the code NCT03504917.

**Supplementary Information:**

The online version contains supplementary material available at 10.1038/s41598-025-22659-y.

## Introduction

The autism spectrum disorder (ASD) is defined in the Diagnostic and statistical manual of mental disorders (5th ed., text rev.)^1^ by challenges in social communication and interactions (CSCI), restricted and repetitive behaviors and interests (RRB), and differences in sensory processing. Alongside these core features, autistic people often experience mental health problems such as anxiety, depression, sleep deficits, and attention deficit hyperactivity disorder^2^. Autistic adults often undergo treatment by healthcare providers who are unfamiliar with the unique clinical presentation of these disorders in autism, increasing the burden imposed by co-occurring mental health problems^3^. Among these, anxiety is especially pervasive^4–6^, with the current prevalence of a diagnosed anxiety disorder estimated at 27%^7,8^. In addition, multiple studies have found elevated levels of trait anxiety in autistic children and adults when compared with neurotypicals^9^. In adults, one study found elevated symptoms of social anxiety (45% vs. 27%) and trait anxiety (44% vs. 36%) in autistic people compared with neurotypicals^10^.

While the co-occurrence of anxiety and ASD is well established, the relationship between symptom severities has been examined in fewer studies^11–19^ (see Supp. Table 1) and only once in adults^20^. These studies have not provided a definitive picture of the relationship between anxiety and RRB and CSCI. Indeed, whereas seven out of nine studies found a significant relationship between RRB and anxiety^11,13,15,17–20^, the picture is less clear in the case of CSCI and anxiety, as four studies^11,12,16,18^ found a positive and significant relationship, compared with three studies^14,15,20^ that found no significant correlation.

In adults the association between anxiety and core ASD symptoms remains largely under-researched. A single study^20^ examined quantitatively the interaction between these three symptom domains in a cohort of 742 adults and found a positive association between anxiety and RRB, but not between anxiety and CSCI. Qualitative research has suggested that RRB can, but do not need to be linked to anxiety^21–23^.

The paucity of quantitative research in the association between symptoms in autistic adults is particularly problematic, as clinical evidence points to changes in the clinical profile of RRB throughout the lifespan of autistic people. RRB are often divided into low-order RRB, which encompass sensory-motor behaviors such as hand flicking or rocking, and high-order RRB, which involve restricted and intense interests or exaggerated adherence to routines^24,25^. High- and low-order RRB have been reported to undergo different developmental trajectories, with low-order RRB decreasing over time and high-order RRB maintaining a more stable trajectory^26,27^. This pattern has not always been replicated^28^.

It is important to note that RRB are a complex core feature of autism, which, from the lived experience of autistic people, have both positive and negative impacts, sometimes mediated by the evaluation of those around autistic people^21^. Correspondingly, RRB should not be considered intrinsically problematic, especially when deemed positive by people with autism and not associated with self-injury.

Understanding the relationship between anxiety and RRB and CSCI could support development of novel treatments and services for some of the challenges that autistic people face. For example, if the relationship is confirmed, ameliorating anxiety in ASD might help some individuals struggling with complex social contexts^29^, or might reduce certain specific RRB triggered by anxiogenic situations^30–32^. Thus, more research on the relationship between anxiety and ASD is urgently needed, especially in adults.

The goal of this study is to investigate the association between anxiety, CSCI and RRB symptom severity in V1aduct (NCT03504917), a recent, large, Phase 3 clinical trial. V1aduct was designed to test the efficacy of balovaptan^33^, a selective antagonist of the vasopressin 1a receptor, in improving social communication in autistic adults. After a scheduled interim analysis, V1aduct was stopped as it was unlikely to reach its primary endpoint and main results were published^34^. Despite this negative result, V1aduct offers a unique opportunity to investigate the clinical presentation of anxiety in a large and well-characterized cohort of autistic adults over the course of up to 52 weeks. We evaluated longitudinal data collected over the course of the trial, as well as baseline clinical scores. Data from both treatment and placebo arms were pooled together and analyzed blindly due to the lack of treatment effects in the scheduled interim analysis in V1aduct.

## Methods

### Procedure

V1aduct was a double-blind, placebo-controlled, parallel-group, 24-week, Phase 3 study conducted between August 2018 and March 2020^34^. Thus, there was a small overlap between study conduct and the COVID-19 pandemic. Participants were randomized (1:1) to either balovaptan (10 mg) or placebo daily. After 24 weeks, participants could enroll in a 104-week open-label study. Among the inclusion criteria were a Diagnostic and Statistical Manual of Mental Disorders, Fifth Edition (DSM5)-based diagnosis of ASD confirmed by the Autism Diagnostic Observation Schedule 2 (ADOS-2)^35^, a Social Responsiveness Scale (SRS)^36^ total *t*-score above 66, intelligence quotient (IQ) above 70, as well as stable pharmacologic and behavioral treatment throughout the study. Patients receiving medications thought to interfere with the metabolism of balovaptan or to have confounding efficacy effects were excluded from the study. V1aduct was conducted across 46 research sites located in Europe and North America. The study was conducted in full accordance with the Declaration of Helsinki and the International Council for Harmonization E6 guidelines for Good Clinical Practice, or the relevant local laws and regulations. Ethics approval was obtained before study initiation from the corresponding institutional review board or ethics committee.

Participants were first invited to a screening session at which informed consent was obtained and the autism diagnosis was confirmed based on the ADOS-2. The clinical visits took place at baseline (Week 1) and at Weeks 8, 12, 24, 32, and 52, during which the clinical instruments reported here were administered.

### Measures

#### Primary and secondary endpoints

As previously reported^34^, the primary endpoint of V1aduct was change from baseline in the Vineland-II DC at week 24 evaluated in the intention-to-treat population. The Vineland-II DC is the mean of the Social and Communication subdomains, and has been used in other clinical trials in ASD^37,38^. Secondary endpoints were: change from baseline of the Vineland-II DC at week 12, change from baseline of the Pediatric Quality of Life Inventory version 4.0 on summary and total score on weeks 12 and 24; change from baseline in the Vineland-II Adaptive Behavior Composite Standard Score at weeks 12 and 24; change from baseline on the Vineland-II Socialization Domain Standard Score at weeks 12 and 24; change from baseline on the Vineland-II Communication Domain Standard Score; Change from Baseline in Vineland-II Daily Living Skills Domain Standard Score at weeks 12 and 24; change from baseline and improvements in Clinical Global Impression severity at weeks 12 and 24; change from baseline in the Hamilton Anxiety Rating Scale total and domain scores at week 12 and 24; proportion of subjects with an improvement higher than 6 points in the Vineland-II DC Score; and the percentage of participants with adverse events at week 24 and after 2 years. All endpoints were analyzed by means of ANCOVA with baseline as covariate and geographical location, age group, sex and treatment group as fixed effects.

The main goal of the current study is to investigate the correlation between anxiety, RRB, and CSCI. Hence, we focused on three clinical scales: The Hamilton Anxiety Rating Scale (HAM-A), RBSR, and the VABS-SC. In addition, we report the Wechsler Abbreviated Scale of Intelligence Full Scale IQ (WASI).

#### HAM-A

The HAM-A (Hamilton Anxiety Rating Scale)^39^ is a clinician-administered instrument designed to measure anxiety severity. It is not a diagnostic instrument, and it does not distinguish between different anxiety disorders such as social anxiety, panic disorder or generalized anxiety disorder. Moreover, while it is a widely used instrument in clinical research, its validity in ASD has never been investigated. The HAM-A consists of 14 items scored from 0 to 3, covering mood, psychosomatic, and cognitive domains. Higher scores indicate more accentuated signs of anxiety. A total score above 17 indicates mild-to moderate or moderate-to-severe anxiety.

#### RBSR

The RBSR (Repetitive Behaviors Scale – Revised)^40,41^ is an informant-based, 44-item scale designed to measure RRB in ASD. Each item is scored on a Likert scale from 0 to 3 according to the frequency and severity of the behavior. Higher scores indicate more prominent RRB. The RBSR sees use both in pediatric and adult populations. It comprises six subscales^40^: Restricted, stereotypic, self-injurious, compulsive, sameness, and ritualistic behaviors. The first three are associated with low-order RRB, whereas the latter three are related to high-order RRB^24^.

#### VABS-SC

The VABS (Vineland Adaptive Behavior Scales 2)^42^ is a clinical instrument divided into four domains: Communication, Daily Living Skills, Motor Skills, and Socialization. Items in these domains are scored according to their frequency. This instrument is widely used in clinical research on adaptive behavior in children and adults^43^. We report here the mean of the Socialization and Communication subdomains (VABS-SC), which assesses behaviors associated with CSCI. The VABS is clinician rated and caregiver reported. Higher scores indicate greater adaptive behavior.

#### WASI

The WASI (Wechsler Abbreviated Scale of Intelligence Full Scale IQ)^44^ is a widely used instrument to measure fluid intelligence in individuals between 6 and 85 years of age. It was used to include only individuals with no intellectual disability (IQ > 70).

### Randomization

Participants were randomized using permuted blocks of 4 through an independent voice or web system. The system assigned a blinded kit numbers of either balovaptan or placebo. Randomization was provided by Signant Health. The sponsor of the trials did not have live access to the randomization sequence. Participants, sites, and the sponsor were blinded to treatment assignment during the trial.

### Statistical methods

The sample size of V1aduct (*N* = 350) would have provided 85% power to detect a difference of 4 points in change from baseline in the primary end point (Vineland-II DC at week 24) between the placebo and balovaptan groups, assuming a standard deviation of 12.5 points and a significance level of α = 0.05.

An interim futility analysis was conducted by an independent committee when approximately half of the planned number of participants (175) had completed the week 24 visit.

To analyze the correlation between anxiety, RRB, and CSCI, we fitted mixed effect models to each outcome measure. Age, sex, IQ, use of any medication to treat anxiety or depression, and weeks from baseline of the clinical visit in the form of a second order polynomial (visit and visit**2) were entered as fixed effects. Participants were entered as random intercepts. Pearson’s correlations across outcome measures were computed from the random intercepts associated with each participant and scale. This analysis removes the effect of confounding factors like IQ and sex and is comparable to a partial correlation analysis. Baseline data were analyzed similarly, first regressing out the effects of sex, age, IQ, and use of any drug against depression or anxiety on each outcome. We then extracted the residuals and computed their Pearson’s correlation. As we tested for correlations between HAM-A, RBSR, and VABS-SC, we Bonferroni corrected for multiple comparisons setting α = 0.05/2 = 0.025.

The present analysis was blind to the treatment arm and, therefore, we did not investigate any possible pharmacologic effect of balovaptan. We opted not to account for the treatment arm in our analysis because balovaptan did not show any significant effect on V1aduct’s primary endpoint (VABS-SC)^34^. Moreover, since the study was primarily powered to detect differences between treatment arms in the primary endpoint, given the early termination of the study, we refrained from post-hoc evaluations of differences in clinical endpoints across treatment arms. Nevertheless, we also report comparable analyses on data from the baseline visit to test if the same conclusions could be reached when only considering data from before treatment onset.

## Results

**Fig. 1:**
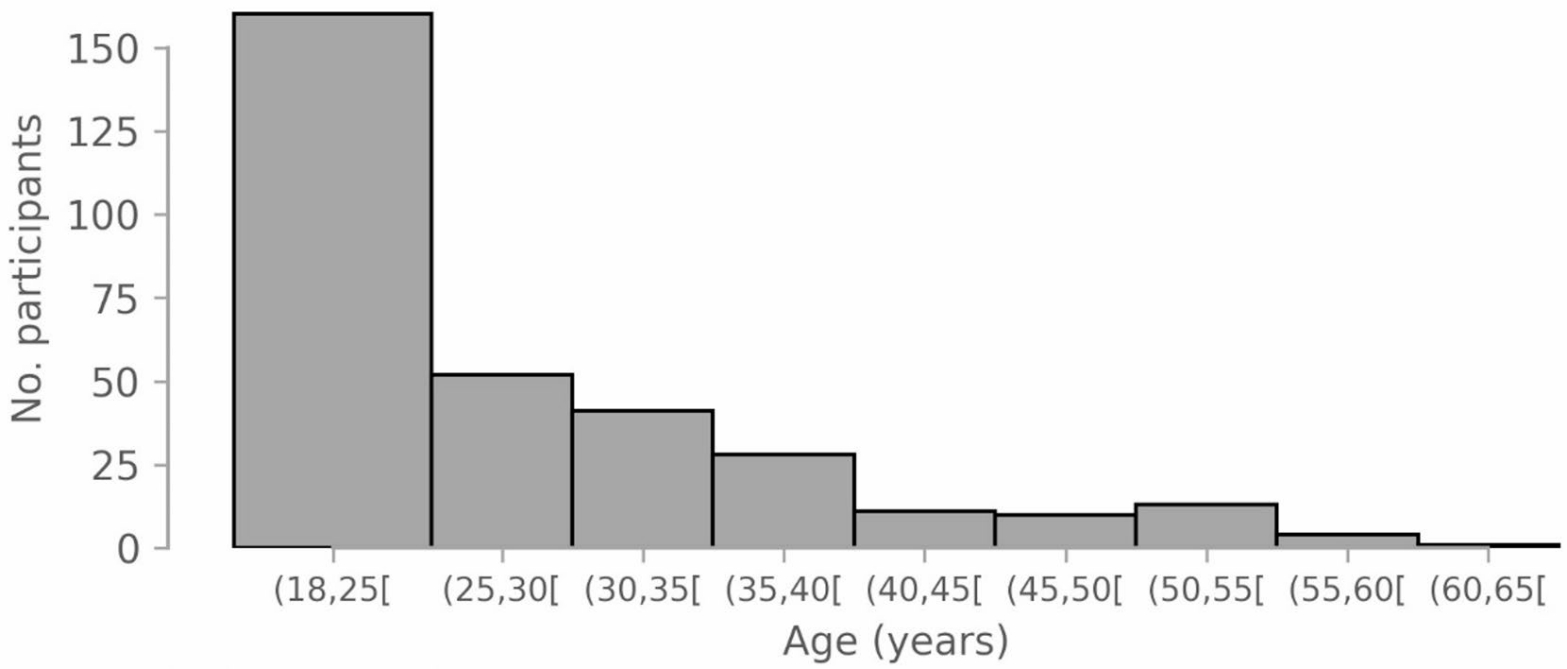
Participants’ age distribution.

Before V1aduct was terminated, 322 participants (64 females, mean age 27 ± 10 years, range: 18–62) were recruited and randomized. The study flow can be found in ^34^. Table 1 displays key demographic characteristics at baseline. More detailed demographic information about the sample has been reported elsewhere^34^. We included data from the open-label extension study, when available. One participant was excluded because they were enrolled but no visit was completed before V1aduct was stopped. Males were overrepresented (258) compared with females (64), reflecting previous estimates of the prevalence and historical rates of ASD in the general population. Importantly, as shown by Fig. 1, young adults were overrepresented in our sample, likely reflecting the historical under-diagnosis of ASD in adult populations^45^.

**Table 1:** Key demographic information.

	Balovaptan group (*N* = 163)	Placebo group (*N* = 158)
Age years		
Mean (SD)	27.6 (9.7)	27.6 (9.8)
< 25	82 (50%)	81(51%)
≥ 25	81 (50%)	77 (49%)
Sex		
Female	35 (21%)	29 (18%)
Male	128 (79%)	129 (82%)
Geographical region		
North America	131 (80%)	129 (82%)
Rest of the world	32 (20%)	29 (18%)

Treatment groups were pooled for the present analysis.

At any point during the study, seventy-four participants received medication to treat anxiety or depression, such as benzodiazepines or selective serotonin reuptake inhibitors. Treatment for anxiety or depression was incorporated as two independent categorical covariates in the main statistical analyses.

After the interim futility analysis, V1aduct was stopped because it was deemed unlikely to reach its primary endpoint and thus, neither primary nor secondary endpoints were evaluated for statistical significance. Point estimates are reported in ^34^. Adverse events encountered during the double-blind treatment period are also reported in ^34^.

**Fig. 2:**
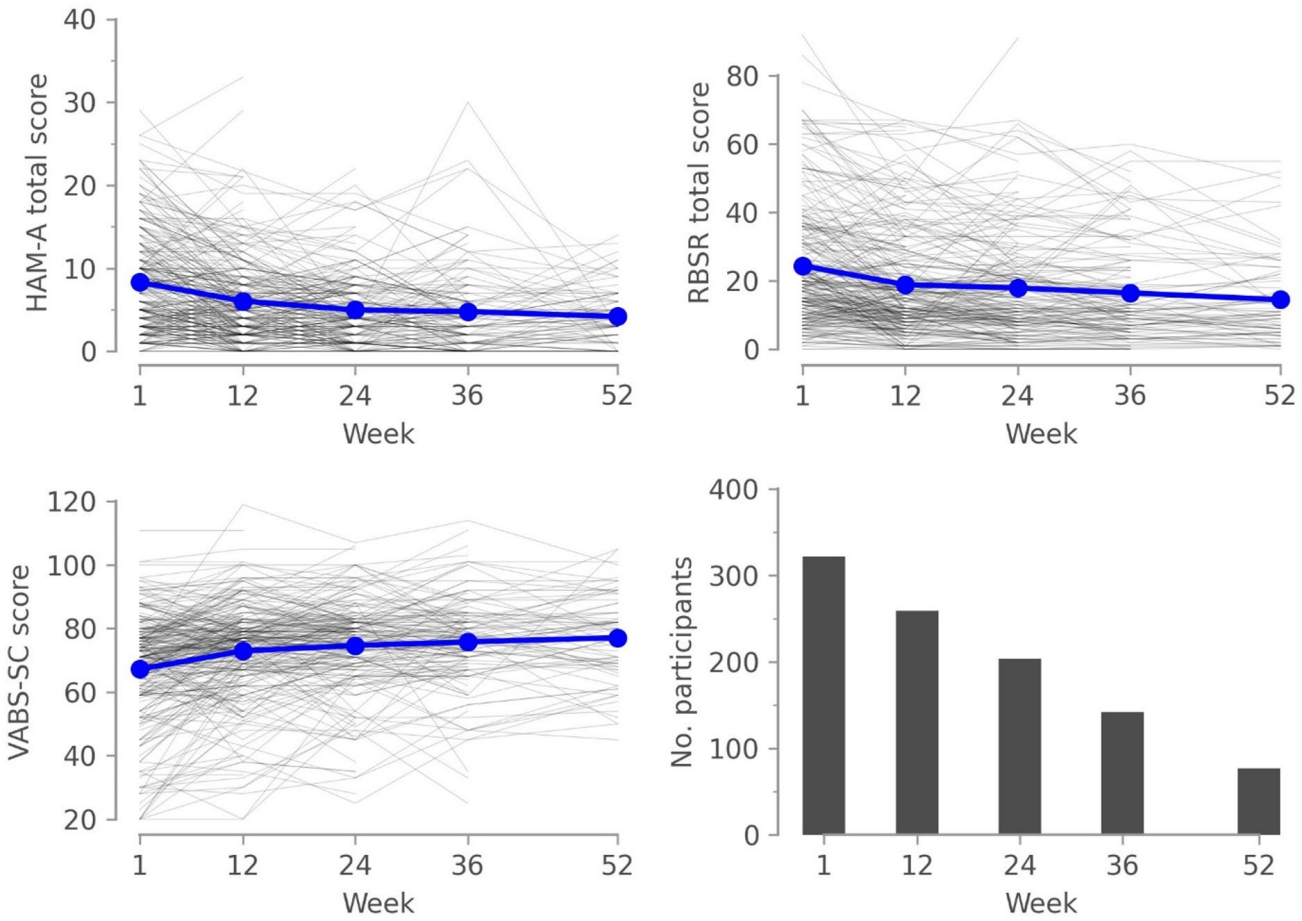
Clinical Outcomes as a Function of Study Week. Gray lines represent the scores of individual participants. Blue solid lines represent the mean values. HAM-A = Hamilton Anxiety Rating Scale, RBSR = Repetitive Behaviors Scale – Revised, VABS-SC = Vineland Adaptive Behavior Scale 3rd Edition, Socialization & Communication Domains standard score.

All the following analyses reported here were not pre-specified and are exploratory in nature. Figure 2 displays the scores of the clinical scales collected from Week 1 to 52 and the number of participants with data available at each visit. A small number of participants (41, 12%) had a HAM-A score above 17 at any point during the study, indicating at least mild-to-moderate anxiety symptoms.

For all three outcome measures, there was a large improvement in symptom severity at the start of the study, after which changes in the clinical symptoms tended to plateau, especially after Week 24. This was captured by the significant quadratic terms associated with the visit day (visit**2) in each model (see Table 2).

Female participants displayed higher anxiety levels than males (*t* = –3.6, *P* < 10^–3^ ) throughout the study. Those who were treated for anxiety (*t* = 2.9, *P* = 0.004) or depression (*t* = 2.6, *P* = 0.009) had on average higher HAM-A scores, but this difference was small, amounting to less than 2 points in the scale. Males had higher RBSR scores than females (*t* = 2.8, *P* − 0.006).

While RRB tended to decline with age, RBSR differences were not significant (*t*=–1.9, *P* − 0.055). Since previous studies have reported that high- and low-level RRB follow different developmental trajectories, we considered each of these two subdomains of the RBSR independently. Low but not high-order RRB were negatively correlated with age at an uncorrected α = 0.05 (*t*=–2.4, *P* = 0.018; see Supp. Table 2), although this relationship was small with a decrease of 0.7 points per decade. No correlation was found between IQ (WASI) and RRB (HAM-A; t = 1.3, *P* = 0.178). No association was found between age and RRB.

IQ had a significant impact on the VABS-SC (*t* = 3.5, *P* < 10^–3^), with individuals with higher IQ showing higher scores. Males displayed lower social interaction and communication scores than females (*t* = 3.5, *P* < 10^–3^). There was a significant (*t* = 2.4, *P* = 0.016) but small negative effect of age on scores in the VABS-SC, with a decrease of 2 points in the scale each decade.

**Table 2:** Estimated models of all clinical scores.

Variable	Estimate	t value	*P*
HAM-A			
Intercept	4.4	2.6	0.009
Visit	–37.0	–9.8	< 0.001
Visit**2	19.2	5.4	< 0.001
Sex—male	–2.3	–3.6	< 0.001
Age	0.1	2.3	0.023
Anxiety med.	1.8	2.9	0.004
Depression med.	1.7	2.6	0.009
IQ	0.0	1.0	0.336
RBSR			
Intercept	21.5	3.9	< 0.001
Visit	–91.5	–10.7	< 0.001
Visit**2	36.0	4.6	< 0.001
Sex – male	5.9	2.8	0.006
Age	–0.2	–1.9	0.055
Anxiety med.	1.8	0.9	0.383
Depression med.	–0.8	–0.4	0.722
IQ	0.0	–0.3	0.740
VABS-SC			
Intercept	60.4	11.6	< 0.001
Visit	94.5	9.6	< 0.001
Visit**2	–37.6	–4.1	< 0.001
Sex – male	–4.5	–2.3	0.025
Age	–0.2	–2.4	0.016
Anxiety med.	0.0	0.0	0.987
Depression med.	–2.2	–1.1	0.293
IQ	0.2	4.5	< 0.001

Visit = Week in which the corresponding clinical visit occurred. Visit**2 = Second order polynomial of visit. HAM-A = Hamilton Anxiety Rating Scale, IQ = Intelligence quotient, med. = Medication, RBSR = Repetitive Behaviors Scale – Revised, VABS-SC = Vineland Adaptive Behavior Scale 2, Socialization and Communication Domains.

**Fig. 3:**
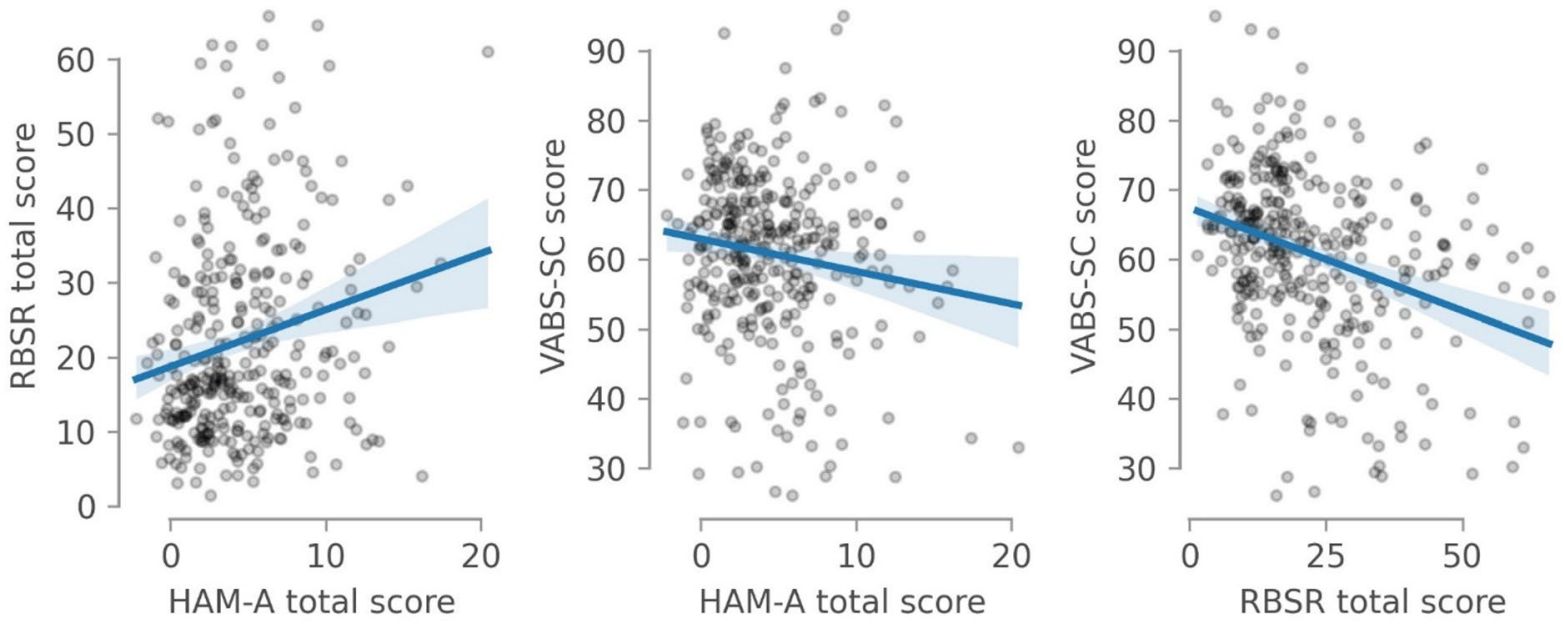
Relationship Between HAM-A Scores and Other Clinical Scales. Correlation between HAM-A, RBSR and VABS-SC scores after accounting for age, IQ, and day in the study. Solid lines represent the estimated correlation and shaded areas represent 95% confidence intervals. HAM-A = Hamilton Anxiety Rating Scale, IQ = Intelligence quotient, RBSR = Repetitive Behaviors Scale – Revised, VABS-SC = Vineland Adaptive Behavior Scale 2, Socialization and Communication Domains.

Most central to our analysis, we examined the correlation between anxiety and RRB and CSCI. In congruence with most previous studies, there was a significant correlation between HAM-A and RBSR scores (Fig. 3, *r* = 0.22, *P* < 10^–3^), indicating that anxiety and RRB are associated with each other in adults with ASD. Moreover, there was also a smaller association between anxiety and CSCI represented by a significant correlation between HAM-A and VABS-SC (Fig. 3, *r* = –0.16, *P* = 0.007).

**Fig. 4:**
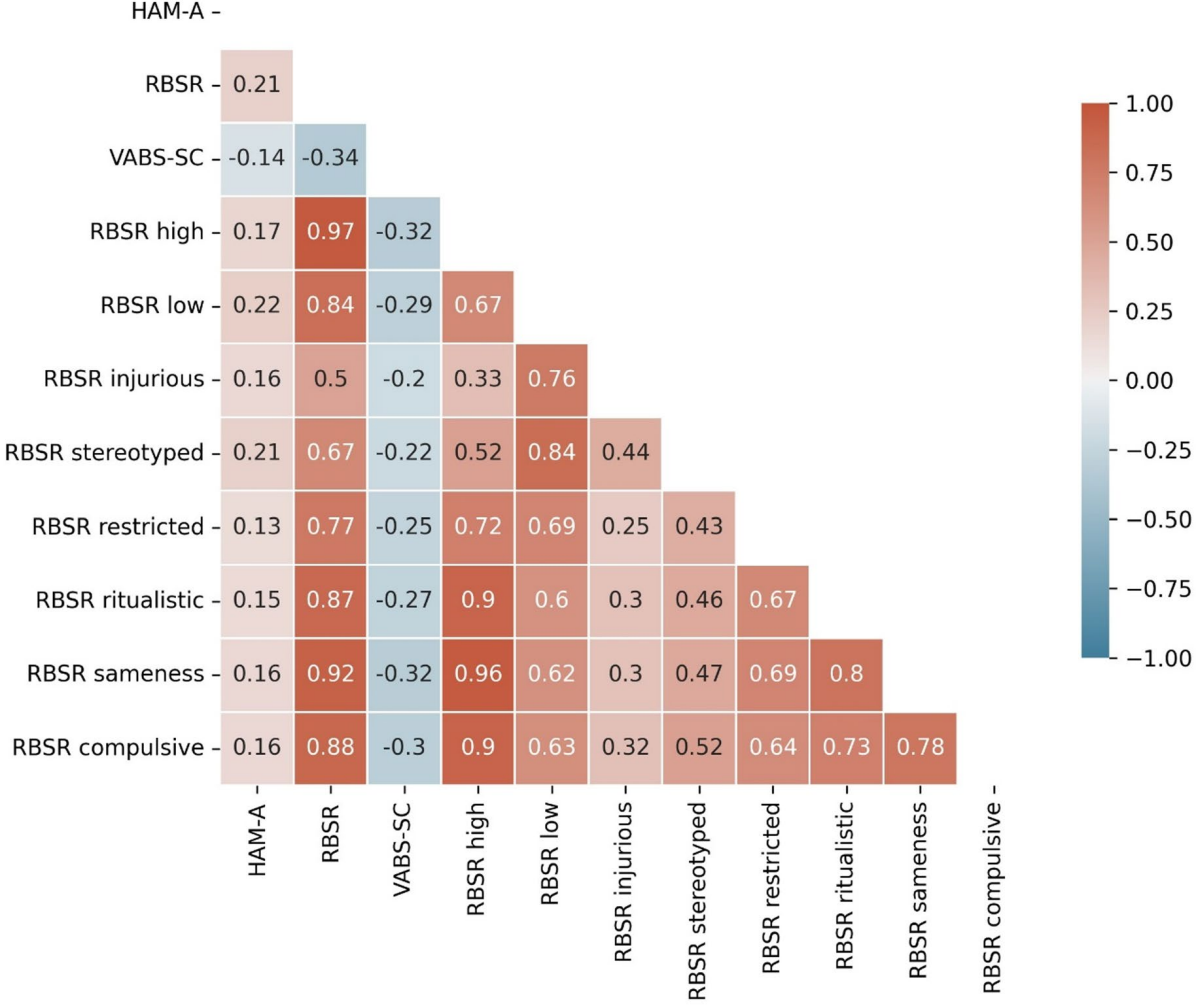
Correlation coefficients between HAM-A, VABS-SC and RBSR scores and its subscales. Correlation between HAM-A, VABS-SC, RBSR scores and subscales after accounting for age, IQ, and day in the study. HAM-A = Hamilton Anxiety Rating Scale, IQ = Intelligence quotient, RBSR = Repetitive Behaviors Scale – Revised, VABS-SC = Vineland Adaptive Behavior Scale 2, Socialization and Communication Domains.

Regarding the subscales of the RBSR, both low- and high-order RRB domains were significantly correlated with anxiety (*r* = 0.22, *P* < 10^–3^, and *r* = 0.17, *P* = 0.002, respectively). We also considered the correlation between the HAM-A and each of the six individual subscales of the RBSR as shown in Fig. 4. The *Stereotyped Behaviors* sub-scale had the strongest correlation with the HAM-A (*r = 0.21*) while other subscales had an *r < 0.17*. The *Self-injurious behavior* subscale was not strongly associated with the HAM-A score (*r = 0.16*), comparable to the *Sameness Behavior* and *Compulsive Behavior* subscales.

As a control analysis, we estimated the correlation between scales from the means across visits, as opposed to the random intercepts estimated from mixed effect models. Thus, this control analysis does not correct for any covariate such as age or IQ. In this analysis, the same associations were found to be significant, although slightly larger (HAM-A and RBSR *r* = 0.25, *P* < 10^–3^; HAM-A and VABS-SC *r* = –0.20, *P* < 10^–3^).

In a second control analysis, we included only data from the baseline visit. As demonstrated by Table 3, we found the same relationships, demonstrating that these associations were not caused by participants’ experiences in the clinical trial or by the treatment they received.

**Table 3:** Correlation between clinical scales at baseline.

Variables	*r*	*P*
HAM-A ~ RBSR	0.19	< 10^–3^
HAM-A ~ VABS-SC	–0.13	0.014
RBSR ~ VABS-SC	–0.33	< 10^–5^

Only 319 participants were included in this analysis, as two participants lacked the RBSR in their first visit. HAM-A = Hamilton Anxiety Rating Scale, RBSR = Repetitive Behaviors Scale – Revised, VABS-SC = Vineland Adaptive Behavior Scale 2, Socialization and Communication Domains.

**Fig. 5:**
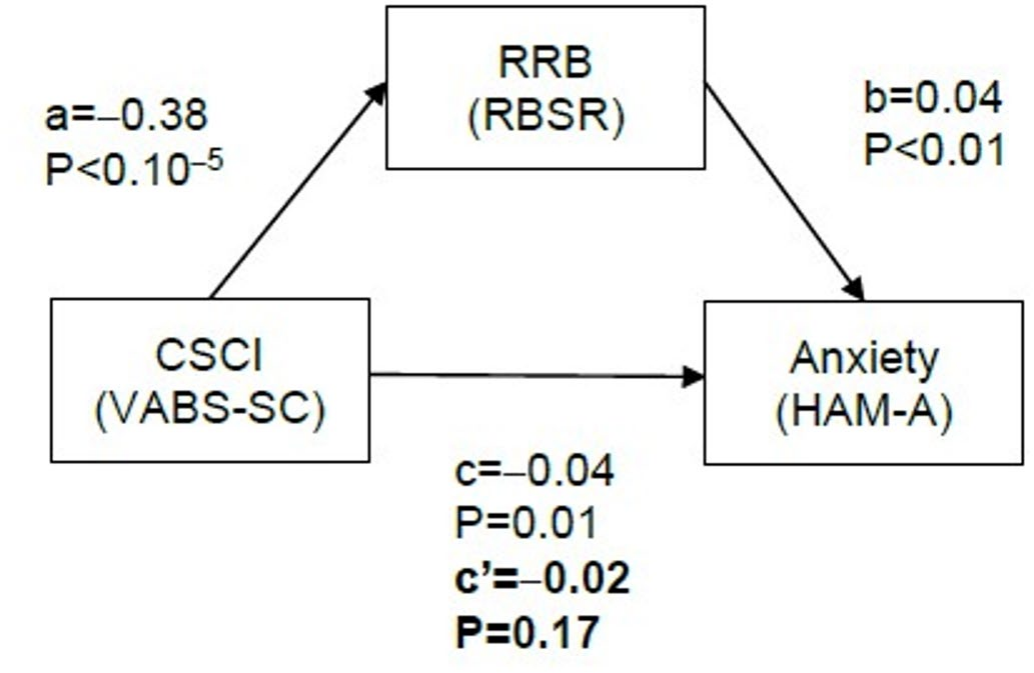
Mediation analysis between clinical outcomes. The effect of CSCI on anxiety was mediated by its effect on RRB. c’ represents the average direct effect of CSCI on anxiety. CSCI = Challenges in social communication and interaction, HAM-A = Hamilton Anxiety Rating Scale, RBSR = Repetitive Behaviors Scale – Revised, RRB = Restricted and repeated behaviors and interests, VABS-SC = Vineland Adaptive Behavior Scale 2, Socialization and Communication Domains.

Although not part of our initial interest, we also considered the relationship between RBSR and the VABS-SC. There was a significant and strong correlation between both outcome measures (*r*=–0.38, *P* < 10^–5^), indicating that larger deficits in social communication were associated with stronger RRB. Since this correlation could drive the association between CSCI and anxiety, we performed a mediation analysis (Fig. 5), to assess if the association between CSCI and anxiety was explained by their mutual relationship to RRB. Indeed, we found that the average direct effect of CSCI on anxiety was not significant (c’=–0.02, *P* = 0.17). Thereby, despite the face-value correlation between anxiety and CSCI, this relationship was likely explained by the strong correlation between RRB and CSCI, and completely disappeared once we controlled for RRB.

## Discussion

The main finding of the current study is the positive association between anxiety and RRB, which demonstrates that not only are anxiety symptoms highly prevalent in autistic adults^7,8^, but also that more pronounced RRB are accompanied by higher anxiety levels. This finding is congruent with the study by Kuzminskaite et al.^20^, who reported a similar association between anxiety and high- and low-order RRB despite using a different set of instruments (self-reported Hospital Anxiety and Depression Scale – Anxiety Component and the Adult Routines Inventory). Other differences between V1aduct and Kuzminskaite’s cohort, a subsample of the Netherlands Autism Register, were a much larger number of females (V1aduct ~ 20% vs. ~ 60%), the use of self-administered instruments, and a higher mean age (27 vs. 43 years). Yet, the findings in both studies are congruent, and convey a similar picture as the pediatric literature (see Supp. Table 1).

A second important conclusion in our study is that the association between anxiety and CSCI is completely mediated by RRB. Interestingly, in a series of hierarchical models Kuzminskaite et al. found that the association between anxiety and CSCI disappeared after accounting for high- and low-order RRB in their regression model. Moreover, there was a stronger relationship between CSCI and high-order RRB (*r =* 0.54*)* than in V1aduct (*r =* 0.17*)*. Considered altogether, the evidence suggests that in the absence of pronounced RRB, elevated social and communications challenges are not associated with anxiety in autistic adults.

In the present study, the association between RBB and anxiety remained significant when we considered high- and low-order RRB independently. Interestingly, the correlation was numerically stronger with low compared to high RRB. Moreover, the correlation between high-order RRBs and anxiety was similar in magnitude to the correlation between the RBSR *Self-injurious Subscale* and anxiety. This suggests that anxiety is not particularly strongly associated with self-injurious RRBs. However, as noted before, the correlations between subscales of the RBSR and the HAM-A were overall small in magnitude.

In our primary analysis, we did not consider treatment arm as a covariate because the futility analysis that led to the early termination of the study revealed that a positive effect of balovaptan was highly unlikely. The early termination of the study also implies that the final sample size was not adequate to detect a difference between treatment arms. Importantly, while our primary analysis was based on longitudinal data, baseline data led to similar conclusions as our main analysis, implying that our conclusions are not affected by the inclusion of all treatment arms.

The existence of a causal relationship between anxiety and RRB and its directionality remain open questions. Several qualitative studies have proposed that RRB have a soothing effect and are a strategy to cope with anxiogenic situations^21–23^. Unfortunately, the current study cannot shed light on the directionality of this relationship. Whatever the causal direction might be, the association between these two symptoms was small in our study (*r* = 0.22) and its clinical significance remains unclear. Importantly, such a small correlation could suggest that even in the presence of a causal relationship between anxiety and RRB, only a very large therapeutic effect on anxiety might have an observable effect on RRB. More generally, whether providing treatment for anxiety disorders in autistic adults could reduce RRB remains unclear. However, the weakness of the correlation reported here does not strongly support the rationale for such an approach.

While Kuzminskaite et al.^20^ appears to have over-sampled females compared to autism prevalence rates, the current study’s 4:1 male to female ratio may not align with current estimates of autism prevalence across the sexes—generally estimate at around 3:1^46,47^. In the present study, because females exhibited higher levels of anxiety than males, it is possible that the findings were impacted by the lower female to male ratio. Future work with fuller representation of females is needed to conclusively address any sex-related differences in the relationship between anxiety and RRB in autism.

A large previous study^11^ indicated that high IQ is correlated with elevated anxiety in children with ASD. In our adult cohort with IQ above 70, we found no evidence of a correlation between IQ at screening (WASI) and HAM-A scores (*t* = 1.3, *P* = 0.178). There was also no significant association between age and the RBSR total score. However, when we considered the low- and high-order RRB, there was a small, yet significant decline in the intensity of low-order RRB with age (–0.7 points per decade). There was no significant change in high-order RRB with age, in accordance with previous reports^26,27^.

Longitudinally, there was an improvement in all scales reported here over the course of the study, regardless of treatment arm. This is not atypical in trials that target affective disorders^48,49^, where improvements have been associated with the placebo effect as well as with the ‘regression to the mean’ phenomenon^50^. While this phenomenon is well documented, its causes are less well understood, and are likely to include true placebo effects, the effect of participating in a clinical trial and the concomitant regular medical monitoring and the regression to the mean phenomenon, by which large deviations from long term trends tend to disappear after enough time.

In the present study, the main measure of anxiety was the HAM-A. This clinical questionnaire measures clinical signs of anxiety and is not comparable to a clinical diagnosis of an anxiety disorder. Moreover, the HAM-A is not designed to distinguish between different anxiety symptoms or anxiety disorders (such as generalized anxiety disorder, social anxiety disorder, specific phobia, etc.). Thus, we cannot address how RRB and CSCI relate to specific forms of anxiety in autism. Nonetheless, prior literature has examined both diverse anxiety disorders or symptoms and social anxiety disorder specifically, in relation to autism spectrum disorder^7,8^, and provides a context for interpreting the present associations, which are based on HAM-A.

The scales employed in V1aduct and reported here were all clinician-assessed or caregiver reported, and thus cannot fully capture the personal experience of participants. This limitation is a consequence of using instruments that target both children and adults with a large variability in their development (i.e. the VABS and the RBSR), as these scales can only be collected with the help of a caregiver with adequate verbal skills. More generally, there is an unfulfilled need to develop and validate clinical instruments that are specific to autistic adults, although some progress has been made in recent years, for example in assessing anxiety^51^.

All six countries that participated in this study are part of North America and Europe. Thus, despite its international character, V1aduct enrolled only participants from developed countries. How and whether anxiety is related to clinical features of ASD in other geographies and socioeconomic contexts remains unexplored in our study and should be addressed in future studies.

V1aduct only recruited adults with moderate or elevated SRS scores and an IQ above 70. Thereby, it cannot provide a picture of how anxiety relates to core ASD features in individuals with an intellectual disability or with milder symptoms. Yet, despite the limitations of the sample reported here, our findings are in good agreement with the literature and indicate that the relationship between anxiety and RRB extends to autistic adults without an intellectual disability.

A further limitation of the present study is that only participants without an affective, psychotic or neurological disorder were included, with the goal of not interfering with the assessment of the study’s endpoints. Since people with autism are at an increased risk of psychiatric disorders, such as anxiety and depression, the study sample is not completely representative of the overall autistic population, and might reflect individuals with less severe comorbidities. This is a possible reason for the relatively low (12%) number of participants with an elevated HAM-A score, defined by the 17 points benchmark. Despite this limitation, our study suggests that the associations between anxiety and the core features of autism observed in pediatric populations are also present in autistic adults with a low burden of psychiatric disorders.

While the present cohort consists of adults from 18 to 62 years of age, most participants were in their 20s. The overrepresentation of young adults likely reflects the historical challenges associated with detecting ASD in adults^4^, and the resulting under-diagnosis of ASD in this population^45^. This may result in a skew of the severity of ASD symptoms in those diagnosed in older age groups and limits the power of the present analysis to detect age-related changes in the clinical presentation of ASD. Nevertheless, we found a small but significant decrease with age in low-order RRB.

Anxiety is a common and pervasive mental health disorder in autistic people and may have a bidirectional causal relationship with RRB and CSCI. Given the large and diverse population included in this study, our analysis provides strong evidence of a small but significant relationship between anxiety and RRB in autistic adults, which closely mirrors previous findings in children and adolescents. Moreover, our study suggests that there is no direct relationship between anxiety and CSCI, and that apparent correlations between these symptoms are mediated by their mutual relation with RRB. These findings could help to better target the clinical interventions and services available to autistic adults.

## Supplementary Information

Below is the link to the electronic supplementary material.

Supplementary Material 1

## Data Availability

The datasets generated and/or analysed during the current study are available in the Vivli repository https://vivli.org/ourmember/roche/.
