# Supplementary material for: Anxiety, repetitive and restricted behaviors and interests, and social communication in autistic adults: an exploratory analysis of a phase 3, randomized clinical trial
Source: Sci Rep. 2025 Nov 6;15:38912. doi: 10.1038/s41598-025-22659-y (PMC12592728; doi:10.1038/s41598-025-22659-y)
Supplement: Supplementary file 1 — Supplementary Material 1 [file 41598_2025_22659_MOESM1_ESM.pdf]

Anxiety, repetitive and restricted behaviors and interests, and social communication in autistic adults: An exploratory analysis of a phase 3, randomized clinical trial

EA Aponte, J Tillmann, T Gleissl, M Del Valle Rubido, L Murtagh, K Sanders, CH Chatham, T Wiese, EE Suter

Study	N	Age group	RRB & Anxiety	CSCI & Anxiety
(Sukhodolsky et al., 2008)	171	Children	Positive	Positive
(Chang et al., 2012)	53	Children	NA	Positive
(Rodgers et al., 2012)	34	Children	Positive	NA
(Hallett et al., 2013)	146	Children	NS	NS
(Magiati et al., 2016)	241	Children	Positive	NS
(Duvekot et al., 2018)	130	Children	NS	Positive
(Baribeau et al., 2020)	421	Children	Positive	NA
(Briot et al., 2020)	79	Children	Positive	Positive
(Ben-Itzchak et al., 2020)	61	Adolescents	Positive	NA
(Kuzminskaite et al., 2020)	742	Adults	Positive	NS

Supp. Table 1: Summary of Previous Studies on the Association Between Anxiety, RRB and CSCI in ASD. Reported significant positive correlations are indicated as *Positive*. CSCI = Challenges in social communication and interactions, NA = Not available, NS = Not significant, RRB = Restricted and repeated behaviors and interests.

Variable	Estimate	*t* value	P
	**RBSR low-order RRB**		
Intercept	6.7	3.5	0.001
Visit	–31.1	–9.5	<0.001
Visit**2	13.1	4.3	<0.001
Sex – male	1.4	1.8	0.069
Age	–0.1	–2.4	0.018
Anxiety med.	0.1	0.1	0.941
Depression med.	–0.2	–0.2	0.826
IQ	0.0	0.5	0.601
	**RBSR high-order RRB**		
Intercept	14.8	3.6	<0.001
Visit	–60.4	–9.5	<0.001
Visit**2	23.8	4.0	<0.001
Sex – male	4.6	2.9	0.004
Age	–0.1	–1.5	0.134
Anxiety med.	1.8	1.2	0.247
Depression med.	–0.6	–0.4	0.696
IQ	0.0	–0.7	0.485

Supp. Table 2: Estimated Models of the Low- and High-Order Domains of the RBSR. P values are nominal and not corrected for multiple comparisons. HAM-A = Hamilton Anxiety Rating Scale, IQ = Intelligence quotient, med. = Medication, RBSR = Repetitive Behaviors Scale – Revised, VABS-SC = Vineland Adaptive Behavior Scale 2rd Edition, Mean Socialization and Communication Domains.

Bibliography

Baribeau, D. A., Vigod, S., Pullenayegum, E., Kerns, C. M., Mirenda, P., Smith, I. M., Vaillancourt, T., Volden, J., Waddell, C., Zwaigenbaum, L., Bennett, T., Duku, E., Elsabbagh, M., Georgiades, S., Ungar, W. J., Zaidman-Zait, A., & Szatmari, P. (2020). Repetitive Behavior Severity as an Early Indicator of Risk for Elevated Anxiety Symptoms in Autism Spectrum Disorder. *Journal of the American Academy of Child & Adolescent Psychiatry*, *59*(7), Article 7. https://doi.org/10.1016/j.jaac.2019.08.478

Ben-Itzchak, E., Koller, J., & Zachor, D. A. (2020). Characterization and Prediction of Anxiety in Adolescents with Autism Spectrum Disorder: A Longitudinal Study. *Journal of Abnormal Child Psychology*, *48*(9), Article 9. https://doi.org/10.1007/s10802-020-00673-0

Briot, K., Jean, F., Jouni, A., Geoffray, M.-M., Ly-Le Moal, M., Umbricht, D., Chatham, C., Murtagh, L., Delorme, R., Bouvard, M., Leboyer, M., & Amestoy, A. (2020). Social Anxiety in Children and Adolescents With Autism Spectrum Disorders Contribute to Impairments in Social Communication and Social Motivation. *Frontiers in Psychiatry*, *11*, 710. https://doi.org/10.3389/fpsyt.2020.00710

Chang, Y.-C., Quan, J., & Wood, J. J. (2012). Effects of Anxiety Disorder Severity on Social Functioning in Children with Autism Spectrum Disorders. *Journal of Developmental and Physical Disabilities*, *24*(3), Article 3. https://doi.org/10.1007/s10882-012-9268-2

Duvekot, J., van der Ende, J., Verhulst, F. C., & Greaves-Lord, K. (2018). Examining bidirectional effects between the autism spectrum disorder (ASD) core symptom domains and anxiety in children with ASD. *Journal of Child Psychology and Psychiatry, and Allied Disciplines*, *59*(3), Article 3. https://doi.org/10.1111/jcpp.12829

Hallett, V., Ronald, A., Colvert, E., Ames, C., Woodhouse, E., Lietz, S., Garnett, T., Gillan, N., Rijsdijk, F., Scahill, L., Bolton, P., & Happé, F. (2013). Exploring anxiety symptoms in a large-scale twin study of children with autism spectrum disorders, their co-twins and controls. *Journal of Child Psychology and Psychiatry*, *54*(11), Article 11. https://doi.org/10.1111/jcpp.12068

Kuzminskaite, E., Begeer, S., Hoekstra, R. A., & Grove, R. (2020). Short report: Social communication difficulties and restricted repetitive behaviors as predictors of anxiety in adults with autism spectrum disorder. *Autism*, 136236132093421. https://doi.org/10.1177/1362361320934218

Magiati, I., Ong, C., Lim, X. Y., Tan, J. W.-L., Ong, A. Y. L., Patrycia, F., Fung, D. S. S., Sung, M., Poon, K. K., & Howlin, P. (2016). Anxiety symptoms in young people with autism spectrum disorder attending special schools: Associations with gender, adaptive functioning and autism symptomatology. *Autism*, *20*(3), Article 3. https://doi.org/10.1177/1362361315577519

Rodgers, J., Riby, D. M., Janes, E., Connolly, B., & McConachie, H. (2012). Anxiety and repetitive behaviours in autism spectrum disorders and williams syndrome: A cross-syndrome comparison. *Journal of Autism and Developmental Disorders*, *42*(2), Article 2. https://doi.org/10.1007/s10803-011-1225-x

Sukhodolsky, D. G., Scahill, L., Gadow, K. D., Arnold, L. E., Aman, M. G., McDougle, C. J., McCracken, J. T., Tierney, E., Williams White, S., Lecavalier, L., & Vitiello, B. (2008). Parent-rated anxiety symptoms in children with pervasive developmental disorders: Frequency and association with core autism symptoms and cognitive functioning. *Journal of Abnormal Child Psychology*, *36*(1), Article 1. https://doi.org/10.1007/s10802-007-9165-9
